# Micro- and nanoplastics concepts for particle and fibre toxicologists

**DOI:** 10.1186/s12989-024-00581-x

**Published:** 2024-04-02

**Authors:** Stephanie Wright, Flemming R. Cassee, Aaron Erdely, Matthew J. Campen

**Affiliations:** 1 Environmental Research Group, School of Public Health, Imperial College London, London, UK; 2 National Institute for Public Health and the Environment, Bilthoven, The Netherlands; 3 Institute for Risk Assessment Sciences, Utrecht University, Utrecht, The Netherlands; 4 Health Effects Laboratory Division, National Institute for Occupational Safety and Health, Morgantown, USA; 5 Department of Pharmaceutical Sciences, College of Pharmacy, University of New Mexico, 87122 Albuquerque, NM MSC09 5360, USA

## Abstract

Micro- and nanoplastic particles (MNP) are omnipresent as either pollution or intentionally used in consumer products, released from packaging or even food. There is an exponential increase in the production of plastics. With the realization of bioaccumulation in humans, toxicity research is quickly expanding. There is a rapid increase in the number of papers published on the potential implications of exposure to MNP which necessitates a call for quality criteria to be applied when doing the research. At present, most papers on MNP describe the effects of commercially available polymer (mostly polystyrene) beads that are typically not the MNP of greatest concern. This is not a fault of the research community, necessarily, as the MNPs to which humans are exposed are usually not available in the quantities needed for toxicological research and innovations are needed to supply environmentally-relevant MNP models. In addition, like we have learned from decades of research with particulate matter and engineered nanomaterials, sample physicochemical characteristics and preparation can have major impacts on the biological responses and interpretation of the research findings. Lastly, MNP dosimetry may pose challenges as (1) we are seeing early evidence that plastics are already in the human body at quite high levels that may be difficult to achieve in acute in vitro studies and (2) plastics are already in the diets fed to preclinical models. This commentary highlights the pitfalls and recommendations for particle and fibre toxicologists that should be considered when performing and disseminating the research.

## Introduction

Micro- and nanoplastic particles (MNP), both granular and fibrous, have been identified in every major region of the planet and in major organ systems of the human body [1]. At present, there is limited evidence to support concerns related to the health impacts of MNPs, but their presence in the environment will double every 10 to 15 years and “dose makes the poison” [1, 2]; thus, the global problem is incredibly complicated and concerning. Notably, particle and fibre toxicologists are uniquely suited to contribute their expertise to help understand the scope of the problem and identify solutions.

The primary route of exposure and uptake of MNPs is likely through the gut rather than air, although certainly there is reason to assess safety of inhaled MNPs [2]. But how the particles absorb, distribute, and potentially accumulate throughout the body is not a simple assessment, and the potential effects on cellular and organ system health also belies simplistic study design approaches. The very physicochemical nature of relevant MNPs requires the expertise of scientists to understand cellular interactions and responses in health and disease. The following commentary highlights a few nuances, challenges, and myths in the study of MNPs to help guide toxicologists as they begin to address this emerging concern.

### Characterization and analysis in matrices

MNPs are not easily measured like other environmental chemicals and specific steps to isolate, process, and visualize are necessary. Furthermore, multidisciplinary approaches and team science are essential to adequately characterize MNP uptake, distribution, and toxicity. Imaging must be complemented with spectral characterization, as from Fourier transform infrared and/or Raman spectroscopy, both of which confidently determine the chemical fingerprint of MNPs, but limitations of size resolution and current libraries of polymer spectra create an unintentional bias of sampling meaning that this approach is only semi-quantitative [3]. While other microscopic methods have been used (i.e., enhanced darkfield microscopy or transmission electron microscopy), these lack the assurance that inclusions have a polymer composition. Even the best imaging spectroscopic instruments can only assess particles as small as 1 μm, leaving nanosized MNPs out of the analysis. Newer models are incorporating improved resolution and integrated spectroscopic techniques (e.g., nano-IR, AFM-Raman, and hyperspectral simulated Raman scattering), and so smaller particle visualization may be possible [3]. But smaller means potentially exponentially more particles, more time intensive analysis, unique expertise, and access to specific instrumentation. Furthermore, it is highly challenging to adequately identify and quantitate MNPs in tissue sections using FTIR, Raman, or electron microscopy due to spectral interference, and often the MNPs need to be isolated from tissues first. Notably, MNPs may have surface features or chemistries that influence biocompatibility, thus visual spectroscopic approaches can be highly informative.

Mass spectrometric methods are highly valuable for quantitation, but important steps must be included to sample preparation. First applied to human blood samples, pyrolysis methods, combined with gas chromatography-mass spectrometry (Py-GC/MS), offer a means of quantifying mass concentration and polymer composition of MNPs [4]. However, for detecting and quantifying MNPs in tissues, digestion or saponification is an essential important step, followed by ultracentrifugation to isolate solids in a pellet form, and exclusion of any chemicals that may be in solution (i.e., styrene) [5]. Py-GC/MS is a very powerful approach to fully identify and quantify the plastics present in tissue samples, although this cumulative assessment of mass polymer concentration does not consider the size of MNPs, which clearly influences toxicity. More advanced separation approaches, including filtration to remove larger MNPs, need to be developed/refined to better address this crucial feature of MNPs.

The above applies to MNP identification, as other methods are available to describe the nature of MNPs in the medium that is used in bioassays. For example, when using suspensions in culture media, size distribution, particle number counts, and even the zeta potential can be measured by using static or dynamic light scattering, differential centrifugal sedimentation, or instrumentation that uses Brownian motion as the main feature of materials in fluids.

### Selection of materials for studies of safety and toxicological mechanisms

“All models are wrong, but some are useful (George E.P. Box)”. Most studies on MNP toxicology to-date have used pristine, polymeric spheres, most commonly polystyrene, which is actually in low proportion relative to polyethylene, nylon, polyvinyl chloride, and other more common plastics to which humans are exposed. Historically, these commercially available particles have been utilized to ask specific questions about the toxicological role of specific physicochemical particle properties, such as size, surface charge, weathering, etc [6]. Application of model polymer microspheres is justifiable for such narrow research questions; however, they do not capture the complex, multifactorial mixture of environmental plastics. Without empirical evidence on the influence of shape and polymer type, which, along with weathered state, crystallinity, hydrophobicity, surface chemistry, and charge, the applicability of the degree of hazard from this model MNP to environmental exposures is unknown. This necessitates that those current conclusions drawn from uniform polymeric spheres should not be extrapolated to represent all MNP exposures. Of course, these can still be used as benchmark materials until proper reference material is widely available, but these should not be used in isolation to explain the potential health impact of environmental MNPs.

To address model MNPs, reference materials– and considerable innovation– is required. The dearth of data on airborne and dietary MNPs in bioavailable size ranges and the absence of measurements of the aforementioned physicochemical properties in environmental studies, raises the question as to what such reference material(s) should comprise. This in part will be driven by the research questions of focus. For example, one could target a key source, such as the MNPs emitted from synthetic textiles, with the understanding this could be further complicated by the choice of fabric (e.g., nylon, polyester, acrylic), application, and therefore diameter of the fibres (and sizes of the particles) or presence and profile of chemical additives. The multidimensionality of environmental MNPs could be simplified, such as by their distribution, but this would not identify problematic attributes, achieved through differential toxicity. Thus, this would not contribute to safer-by-design.

What is clear is that as industry, governmental organisations, and academic research groups engineer their own materials, guidelines for their characterisation are urgently needed. Different ‘synthesis’ methods (e.g., ablating, milling, precipitation) could lead to different particle properties, such as the degree of cross-linking, or the presence of impurities and contaminants, such as heavy or transition metals from milling components. Minimum reporting criteria, including impurities, will ensure comparability and, when the database is large enough, multivariate analyses to distinguish the influential particle properties and whether they come from a particle attribute, or artifact of their production. Until reference materials are available from a centralised source (or sources), at scale, this is one of few solutions.

### State of MNP and sample preparation

There remains a great deal of debate regarding the use of pristine manufactured micro- nanospheres versus environmentally derived or “engineered” MNP models. A role exists for most approaches, but researchers need to be conscious of, and clearly explicate in manuscripts, the caveats for specific models used. Polymer beads/microspheres are excellent models that are highly reproducible, clean of most other contaminants (though they may leach plasticizers), and easily controllable in terms of size and composition. However, the real-world MNP is a heterogeneous mix of size, shape, composition, aging, and surface contaminants [3]. Unfortunately, MNPs in the real-world are difficult to obtain reproducibly, and will need to be processed in the lab to remove endotoxin/pyrogens and other irrelevant toxicants. Weathered, naturally occurring “macroplastics” may provide opportunities, as the friable nature of the aged material may allow milling to micrometer sizes. However, it is very challenging to further transform these to nano-sized particulates. And, batch-to-batch, it will be challenging to ensure a consistent “blend” of MNPs based on composition.

While ingested MNPs can be larger than particles delivered by inhalation, those MNPs that are readily taken up into the body from the intestines are still likely to be smaller than 1–10 μm. Even if larger sized MNP reach the circulation as they will also easily be stuck in capillaries (8 to 10 μm). Innovation in this area of generating relevant MNPs for research is vitally needed. And while reviewers of grants and manuscripts are encouraged to be forgiving to research using engineered polymeric spheres, researchers should pursue novel approaches to better bridge to more environmentally relevant materials.

As evaluated for two decades in the engineered nanomaterial research communities, sample preparation will have a critical effect on issues like stability, size, distribution, as well as the coating of the MNPs with substances otherwise not present in real-world exposure scenarios. Or, the opposite, removal and/or inactivation of chemicals or microorganisms from the MNPs, whether on the surface or from within the MNPs. As a minimal requirement, precise description of sample preparation must be included in a manuscript. In addition, the characterization as mentioned above should also be done on MNPs after sample preparation, preferably in the media that are used to expose cells, tissues, or animals, and related the hypothesis or research question.

### Dosimetrics and dosimetry

**Fig. 1:**
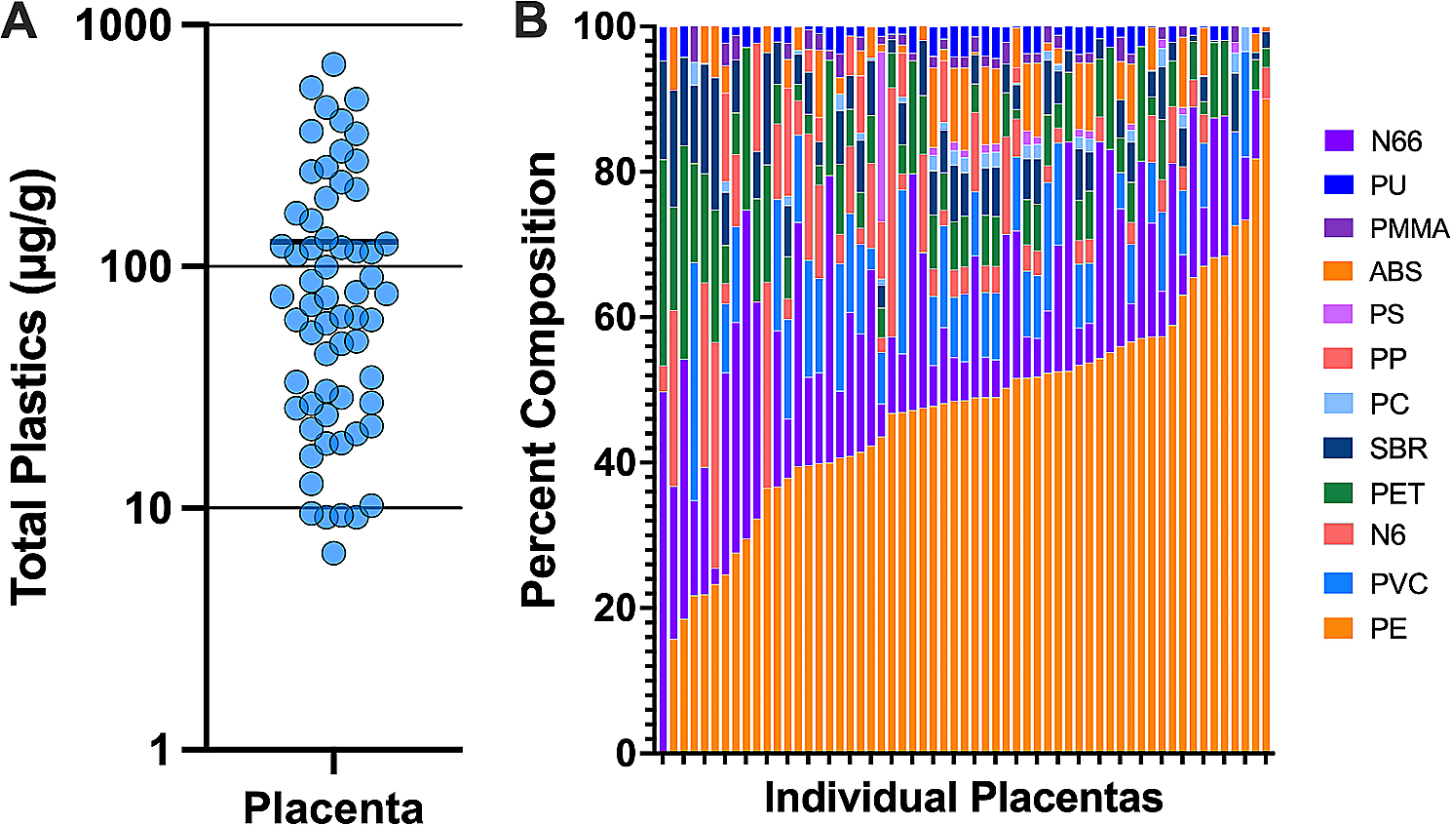
**(A)** Total plastics measured in 62 placentas by pyrolysis-GC/MS (bar reflects mean value). **(B)** Relative distribution of polymers in each placenta sample, illustrating a predominance of polyethylene. Replotted from data presented in [5]. Polyethylene (PE), Polyvinyl chloride (PVC), Nylon 66 (N66), Styrene-butadiene (SBR), Acrylonitrile Butadiene Styrene (ABS), Polyethylene terephthalate (PET), Nylon 6 (N6), Poly(methyl methacrylate) (PMMA), Polyurethane (PU), Polycarbonate (PC), Polypropylene (PP), Polystyrene (PS)

Anecdotally, many in vitro particulate matter toxicity assessments use a mass concentration in media ranging from 3 to 200 µg/ml. We suspect this is largely due to historical work in assessing toxicity of ambient particulate matter, as we have a dearth of information regarding internal dosimetry in humans. While numerous papers have identified the presence of MNPs in various internal organ systems, a true mass concentration estimate (and range) is lacking, which remains a major limitation to the field currently. In blood, a mean level of 1.6 µg/ml was measured, with maximal levels around 10–12 µg/ml [4]. However, in human placentas, which are only approximately 8 months old, levels ranging from 6 to 685 µg/g of tissue weight, with a predominance of polyethylene solids (Fig. 1) [5]; it is very conceivable that other organs systems in adults contain higher concentrations. Hypothetically, MNPs in internal organs may be long ‘lived’, as they are relatively biologically inert.

Acute exposure (hr to a few days) of cell culture exposures may fail to capture the slow exposure dynamics and bias results towards acute in vitro responses. As we are already seeing that some internal tissues contain high concentrations of MNPs, it might seem justifiable to use a dose-response design with high concentrations. But the timing for exposure is quite different in real-life, as the accumulation likely occurs over years. Any particulate exposure > 200 µg/ml is likely to just be overtly toxic to cultured cells, and it is unlikely that such treatments will reveal useful information about the potential health effects of MNPs. In terms of mixtures of MNPs, in vitro models should try to replicate published information on tissue-specific proportions from human or animal studies, and not simply rely on studies of environmental levels, as uptake of specific plastics may be composition dependent and may be based on decades of uptake and historical environmental proportions may not be known. In addition, mass may not always be the ideal metric to describe the concentration-dose-response relationship. Other metrics, such as surface area or particle number counts [3], may need to be considered as it is a known driver of inflammation for certain particulates, especially when comparing various MNPs for their toxic potency.

To increase the value of in vitro model-derived data, efforts should be undertaken to assess the biologically effective dose and dose rate, as low-density materials such as MNPs, effects may be underestimated due to a significant low dose (or even absent in the case of buoyant particles) than assumed by using concentration and cell surface area as the basis for extrapolation. Advanced in vitro models, such as air-liquid interface, upside down cultures where MNPs in culture media are below the cells, or organoid cultures, may be needed if conventional liquid submerged cultures prove ineffective. Where MNPs may differ from other particle dosimetry is in their (low) density. Plastic materials span specific gravities of < 1 to > 1 (∼0.9 to > 2.0/1.8 g/cm^3^ with/without fluoropolymers). Hence, different microplastics of equivalent size will undergo different settling velocities. This is particularly important for in vitro experiments, where conclusions may be drawn in the absence of calculating the delivered dose for different particles. The potential for some of the lower density microplastic to float or remain suspended in denser culture medium needs to be assessed. Whether acquisition of a biomolecular corona increases density over time, resulting in an increase in delivered dose over time is also currently untested [7].

For in vivo models, environmentally relevant dosing is necessary, though confident data on ingestion is lacking. One could easily obtain current estimates in the literature for ingestion, the “credit card per week” has been suggested [8], and yet likely needs revision with data from multiple sources. There may be specific study designs that are irrelevant, like selective exposures in early life or intermittent exposures, as the MNP ubiquitousness means we will always be exposed and exposures are likely getting worse over time. Given that the environmental levels are doubling every 10–15 years [1], it would be relatively justified to say that 8x the current ingestion rate is a model for the year 2070. Notably, standard rodent chow and other sources of food will contain some level of MNPs at baseline, which can be assessed by Py-GC/MS. There is uncertainty in the accumulation rate of MNPs into plants and livestock, and further uncertainty regarding the saturation of uptake mechanisms in the gut, thus researchers would need to be appropriately self-critical of any such conceptualized justifications. This is especially important when justifying a complete “credit card” consumption dose extrapolation and applying that to polymeric spheres of a single type and size. Even more caution should be applied when simply using mathematical equations to incorporate the assumption that all plastic was nano-sized. Research questions regarding dietary influences on MNP uptake, identifying which sources contain the most MNPs or what factors contribute to greater MNP uptake, will be important for understanding risks for the general population.

### Absorption, distribution, metabolism, and elimination (ADME)

We feel that fundamental observations are needed to understand the capacity for an increasing environmental contaminant to render human health effects. It is without question that MNP exposures will continue to grow exponentially for decades regardless of actions taken now. It is important to quickly answer questions as to the accumulation and clearance of MNPs from specific target organs, in both health and disease. All facets of ADME are likely to be much slower than any other toxicant. If retention outpaces clearance, then accumulation curves may need to be derived over years if not decades, which surpasses the utility of conventional preclinical research models. Moreover, testing in humans over decades will be challenging to model given the constant changes in exposure. Although plastics are in general thought to be poorly immunoreactive, the immune system may attempt to metabolize and clear MNPs, which though unlikely to be successful, may lead to chronic immune modulation that can impact disease [9]. Cellular clearance and metabolism may alter the particle surface corona, lead to aggregation of proteins, or become a substrate for non-self signalling [10]. Lastly, the influence of diet, genetics, and disease states on uptake and clearance of MNPs will need to be better detailed, again for all major organs of interest to determine impact.

### Perspectives

MNP exposures have been ongoing for many decades, and we are only now seeing the tip of the iceberg. There are numerous diseases and syndromes that have also been increasing globally in recent decades that are relatively unexplained, including diseases of the gastrointestinal tract, immune system, brain, and reproductive organs; thus, there is justification to consider the role of MNPs in causing or exacerbating those diseases. However, we need information rapidly, rigorously, and from many laboratories.

We should quickly utilize the knowledge gained from the recent decades of particulate matter and engineered nanomaterial research to move forward. Material selection, extensive characterisation with emerging new technology for detection, detailed methodological approach for reproduction by others, multiple particle comparison especially if using polymeric spheres as a surrogate, wider dose ranges, and human exposure relevance are just some of the approaches that should be second nature to particle and fibre toxicologists. In the early years of engineered nanomaterial primary particle research, materials being synthesized could be obtained directly from the company for evaluation [11]. Many of those materials are in production still today while some of the more toxic ones have been substituted. As we gained knowledge of human exposure with time the potential health effects became clearer for engineered nanomaterial classes. For MNPs, the use of polymeric spheres of a single type seems like a step backward compared to the start of engineered nanomaterial research as these materials are continually being challenged as to relevance compared to environmental plastics. Focus needs to be steered to generating a representative study exposure design.

Particle toxicologists have an essential role to play in what could be a potentially major concern to human health and well-being. Our collective experience in handling, characterising, measuring, and determining relevant deposited doses of particles will help shed light on the biological and pathological impacts of these ubiquitous contaminants. As a last perspective, as we expand research endeavours on MNP, the field is encouraged to set aside competitiveness and think broadly about the value and rigor of individual research works as we review grants and manuscripts. The authors of this editorial feel that we will need many hands-on-deck to address the myriad of health and ecological effects of MNPs, and we will need to collectively support the field through rigorous but encouraging peer review.

## Data Availability

Not applicable.
